# Annotations

**Published:** 1910-02-19

**Authors:** 


### ANNOTATIONS.

### The Sale of Proprietary Medicines.

The disproportion between the incomes of proprietary medicine manufacturers and those derived from medical research or from club practices is one of the standing evils from which the profession suffers. A practical remedy too little considered may be found in catering more for the taste and convenience of the public by adopting more thorough methods of pharmacy. Progressive hospital dispensers are now making up leeway in this direction, and providing compounds which formerly could only be obtained on the commercial market, and which, therefore, probably had an uncommonly large and wide sale. In a recent number of the *Berliner Tageblatt* we notice the familiar advertisements of Scott's Emulsion and Hommel's Hæmatogen. The great number of malt extracts offered for sale in England (which analysis has shown to be of very varying value) seem, however, to be replaced by one or two brands of native origin. We are glad that in this country, except now and again, sanatoriums are not puffed in the lay Press. In the journal mentioned a notice of such institutions appears in close proximity to announcements to " Schwache Männer," and the prospectus of " Das intime Buch der Frau."

### Public Health and Quarantine in Australia.

With a coast-line of from 8,000 to 11,000 miles to protect, quarantine is a vital question to Australians. Unfortunately differences of opinion have arisen among public health authorities as to whether or not the Federal Quarantine Act entirely overrides State legislation on quarantine. Pending the settlement of this vexed point, the administration of the Act is virtually suspended, and Dr. Norris, Federal Director of Quarantine, cannot get on with his work. It was the late Dr. D. Astley Gresswell who stirred up both the public and the professional mind to a sense of the dangers which continually confront the community and as to the best modes of prevention, and to him was due the fact that though cases of bubonic plague have been brought to Victoria, the disease has never become endemic in Australia. Rat destruction was, and still is, thoroughly carried out, in conjunction with strict quarantining of cases. An interesting career in connection with public health and quarantine is that of Dr. John S. C. Elkington, who has been appointed to the position of Commissioner of Health in Queensland, lately vacated by Dr. B. Barnett Ham, the present Chairman of the Victorian Board of Public Health. Dr. Elkington, elder son of the Professor of History at the University of Melbourne, and an Australian native, is a graduate of the same University. He spent a year in England, Germany, and Austria studying public health, spent some time working under Dr. Gresswell, whose methods and self-sacrificing spirit influenced him strongly, and for the last six years he has held the position of chief health officer in Tasmania, the island State. There he has brought about a radical change for the better in hygienic conditions. By him the first Australian appointment was made of a medical officer as inspector of school children. Dr. Gertrude Halley (of Melbourne University) was selected for that position about three years ago, and has effected a great deal of good in her classes for the instruction of mothers.

### Operations in two Stages.

In a very thoughtful and timely address to the members of the New York Medical Society, Dr. Howard Lilienthal has lately drawn attention to certain factors in modern operative surgical procedures, the importance of which is too often overlooked. Foremost among the sources of danger to the patient in the more extensive operations now practised are the anæsthetic, the loss of blood, and the shock. Now, all these, but especially the first and most of all the last, depend very largely upon the duration of the operation. All other things being equal, he will get the best results whose technical dexterity and promptness of decision enable him to conclude his operations in the shortest space of time. These are undeniable propositions; but although no surgeon would venture to contradict them, a great many do not in practice act up to them as fully as they should do. The two-stage principle is one which Dr. Lilienthal believes has been too much neglected by surgeons who fear the imputation of timidity, and therefore often run to the opposite extreme of over-boldness. He pleads for a more extensive adoption of the performance of difficult and hazardous operations in two stages, and his list of the operations for which this is recommended includes many which are usually performed at one sitting. Thus, for instance, suprapubic prostatectomy he would perform only some days after a preliminary cystostomy under local anæsthesia; pylorectomy only after a preliminary gastroenterostomy; and excision of the rectum only after a colostomy. The two latter suggestions are not, of course, by any means novel; but probably the bulk of surgeons do these operations in one stage, except when dealing with a particularly enfeebled or exhausted patient. Two more pieces of advice are offered which are quite worth remembering, though necessarily not always practicable. One is to arrange an operation so as to permit cessation at any moment; the other to get through the essential part of the operation early, so that if cessation is necessary the patient will yet have been relieved. Now, all this is very sound sense, as most surgeons will admit, and to force it into notice is an act for which the profession should be grateful to Dr. Lilienthal. The one serious disadvantage to his scheme he unfortunately does not discuss. That is, it is almost needless to say, the reluctance of patients to face the ordeal of the operating table twice. When once they have screwed up their courage to face the terrors of the theatre, their great desire is always to get quite " through with it," and not to have the trying experience of anticipation once more to be faced.

